# Non‐Typhoidal *Salmonella* Colonization in Chickens and Humans in the Mekong Delta of Vietnam

**DOI:** 10.1111/zph.12270

**Published:** 2016-05-06

**Authors:** N. V. Trung, J. J. Carrique‐Mas, N. H. Nghia, L. T. P. Tu, H. H. Mai, H. T. Tuyen, J. Campbell, N. T. Nhung, H. N. Nhung, P. V. Minh, T. T. B. Chieu, T. Q. Hieu, N. T. N. Mai, S. Baker, J. A. Wagenaar, N. T. Hoa, C. Schultsz

**Affiliations:** ^1^ Department of Medical MicrobiologyAcademic Medical CenterUniversity of AmsterdamAmsterdamThe Netherlands; ^2^ Department of Global Health‐Amsterdam Institute for Global Health and DevelopmentAcademic Medical CenterUniversity of AmsterdamAmsterdamThe Netherlands; ^3^ Oxford University Clinical Research UnitCentre for Tropical MedicineHo Chi Minh CityVietnam; ^4^ Centre for Tropical MedicineNuffield Department of MedicineUniversity of OxfordOxfordUK; ^5^ Sub‐Department of Animal HealthMy ThoTien GiangVietnam; ^6^ Preventive Medicine CenterTien GiangVietnam; ^7^ Department of Infectious Diseases and ImmunologyFaculty of Veterinary MedicineUtrecht UniversityUtrechtThe Netherlands; ^8^ Central Veterinary Institute of Wageningen URLelystadThe Netherlands

**Keywords:** Non‐typhoidal *Salmonella*, colonization, antimicrobial resistance, chickens, humans, Vietnam

## Abstract

Salmonellosis is a public health concern in both the developed and developing countries. Although the majority of human non‐typhoidal *Salmonella enterica* (NTS) cases are the result of foodborne infections or person‐to‐person transmission, NTS infections may also be acquired by environmental and occupational exposure to animals. While a considerable number of studies have investigated the presence of NTS in farm animals and meat/carcasses, very few studies have investigated the risk of NTS colonization in humans as a result of direct animal exposure. We investigated asymptomatic NTS colonization in 204 backyard chicken farms, 204 farmers and 306 matched individuals not exposed to chicken farming, in southern Vietnam. Pooled chicken faeces, collected using boot or handheld swabs on backyard chicken farms, and rectal swabs from human participants were tested. NTS colonization *prevalence was* 45.6%, 4.4% and 2.6% for chicken farms, farmers and unexposed individuals, respectively. Our study observed a higher prevalence of NTS colonization among chicken farmers (4.4%) compared with age‐, sex‐ and location‐ matched rural and urban individuals not exposed to chickens (2.9% and 2.0%). A total of 164 chicken NTS strains and 17 human NTS strains were isolated, and 28 serovars were identified. *Salmonella* Weltevreden was the predominant serovar in both chickens and humans. NTS isolates showed resistance (20–40%) against tetracycline, chloramphenicol, sulfamethoxazole‐trimethoprim and ampicillin. Our study reflects the epidemiology of NTS colonization in chickens and humans in the Mekong delta of Vietnam and emphasizes the need of larger, preferably longitudinal studies to study the transmission dynamics of NTS between and within animal and human host populations.

**Impacts**

- We report a higher prevalence of NTS colonization among chicken farmers (4.4%) compared with age‐, sex‐ and location‐matched rural and urban individuals not exposed to chickens (2.9% and 2.0%) in the Mekong Delta of Vietnam.
- This study reports high prevalence of NTS colonization (45.6%) in 204 backyard chicken farms in the Mekong Delta of Vietnam. There was no difference in the NTS prevalence between household‐size farms (10–200 chickens) and small‐size farms (201–2000 chickens).
- Multidrug‐resistant NTS accounted for 27.4% (45/164) of the chicken isolates, 22.2% (2/9) of the farmer isolates and 12.5% (1/8) of the unexposed individual isolates.

## Introduction

Salmonellosis caused by non‐typhoidal *Salmonella enterica* (NTS) is a potentially zoonotic infection commonly associated with gastroenteritis and represents a significant public health problem in both the developing and developed countries (Majowicz et al., 2010).

Although the majority of human NTS cases is the result of foodborne infection (Anon., 2008) or person‐to‐person transmission (Thompson et al., 2013), humans can also become infected with NTS as a result of environmental and occupational exposure to animals, including farm animals (Fone and Barker, 1994; Baker et al., 2007; Hoelzer et al., 2011). This type of exposure is particular prevalent in developing countries, where a large fraction of the population is involved in raising livestock and/or poultry. Farms in these countries are typically backyard or small‐scale and farming procedures mostly involve low levels of biosecurity and personal protection. This results in very close contact between animals and humans. To date, very few studies have investigated the role of exposure to farm animals in asymptomatic NTS infection.

We hypothesized that exposure to chickens through farming results in increased risk of asymptomatic colonization with NTS in Vietnam, a country with a majority of rural population. In this country, small and backyard chicken farming is very common, with most farms typically having less than 50 chickens (Burgos et al., 2007). To investigate this hypothesis, we investigated chicken flocks and farmers to study the prevalence and serovar distribution of NTS organisms. The prevalence in farmers was compared with that among age‐ and gender‐matched individuals living in the same areas but not engaged in poultry farming. In addition, given the extensive antimicrobial drug usage in these chicken farms (Carrique‐Mas et al., 2015), we analysed and compared the frequencies of resistance against key antimicrobial drugs of the most relevant classes, used in both veterinary and human medicine.

## Materials and Methods

### Study population

The target population consisted of chicken farms, including chicken flocks and their farmers, as well as individuals not involved in poultry farming from two rural districts and the capital of the Mekong Delta Province of Tien Giang (Vietnam).

Sampling of farms was conducted at random within two size strata: 10–200 chickens (‘household farms’, *N* = 102) and 201–2000 chickens (‘small farms’, *N* = 102). In selected farms, both flocks and the person responsible for raising the chickens (‘farmer’, *N* = 204) were recruited. In addition to the 204 farmers, 306 participants not involved in chicken or livestock farming were randomly selected (‘unexposed individuals’). A subgroup of unexposed individuals (‘rural subjects’) were selected from the same commune as the farmer and were matched to the farmer by age and gender (*N* = 204, one per farmer). Another subgroup of unexposed individuals (‘urban subjects’) were selected from the provincial capital and were matched to the farmers by age and gender (*N* = 102, one every two farmers).

Farm selection and chicken flock sampling was carried out by staff at the Sub‐Department of Animal Health (SDAH) in Tien Giang. Selection and sampling of human subjects was performed from the population census provided by the Preventive Medicine Centre (PMC) of Tien Giang (Anon., 2007b).

Written informed consents were obtained from all participants prior to participation in the study. Participants that refused to participate were replaced by the next available eligible participant. The study was approved by the Peoples’ Committee of Tien Giang Province, the Department of Health in Tien Giang and the Oxford University Tropical Research Ethics Committee (OxTREC, No. 48/11).

### Sample collection

Farm and human household visits were evenly distributed over a year period from March 2012 to April 2013 in order to avoid seasonal effects. Pooled chicken faeces samples were collected from chicken houses using boot swabs (flocks reared on barn systems) or handheld gauze swabs (caged layer flocks and flocks on stilted mesh houses) as described previously (Nguyen et al., 2015). The sample collection was conducted by a trained sampling team from Tien Giang SDAH.

Rectal swab samples were obtained from all human participants by trained staff from Tien Giang PMC, using Fecalswab (Copan, Italy).

All samples were stored at 4°C, transported to the laboratory at the Oxford University Clinical Research Unit in Ho Chi Minh City and cultured within 24 h after sample collection.

### Laboratory methods

*Salmonella* was isolated using the modification of the ISO 6579:2002 (Annex D) method for chicken faecal samples, involving: (i) pre‐enrichment in 225 ml of buffered peptone water (BPW) (37°C, 18 h); (ii) plating 100 *μ*l of the pre‐enriched culture onto modified semi‐solid Rappaport–Vassiliadis medium (Oxoid; UK) (41°C, 24 h) and (iii) plating onto Rambach agar (37°C, 24 h) (Carrique‐Mas et al., 2009). For human samples, rectal swabs were cultured on MacConkey agar, xylose‐lysinedeoxycholate agar and selenite broth according to the guidelines of World Health Organization (WHO, 2010).

Suspected NTS colonies were selected for each sample and confirmed by slide agglutination with relevant poly O anti‐serum (Anon., 2007a). All isolates confirmed as NTS were further tested for their antimicrobial susceptibility.

Antimicrobial susceptibility testing was performed by disc diffusion method, and breakpoints were interpreted using the Clinical and Laboratory Standards Institute guidelines for Enterobacteriaceae (CLSI, 2011). Eleven antimicrobials were tested including chloramphenicol (30 mg), ceftazidime (30 mg), ceftriaxone (30 mg), amoxicillin/clavulanic acid (30 mg), meropenem (10 mg), ciprofloxacin (5 mg), tetracycline (30 mg), trimethoprim/sulfamethoxazole (1.25/23.75 mg), amikacin (30 mg), gentamicin (10 mg) and ampicillin (10 mg). Quality controls for susceptibility testing and identification were performed every week according to the CLSI guidelines. An MDR strain was defined as a strain resistant to at least three different antimicrobial classes. Chicken farms and farmers were the study unit of analysis. A chicken farm was defined as ‘positive’ for NTS if NTS was isolated from at least one of the boot‐ or handheld gauze swabs.

Confirmed NTS isolates were genotyped using multilocus sequence typing (MLST) as described previously (Achtman et al., 2012). Briefly, pure colonies of overnight culture on nutrition agar were subjected to DNA extraction using Wizard Genomic DNA extraction kit (Promega, Madison, WI, USA). Seven MLST loci (*aro*C, *dna*N, *hem*D, *his*D, *pur*E, *suc*A and *thr*A) were amplified and sequenced in forward and reverse directions using the Big Dye Cycle Sequencing kit (Applied Biosystems, Foster City, CA, USA) on an ABI 3770 automatic sequencer according to the manufacturer's instructions. Sequence data of seven loci were trimmed and blasted to determine sequence type as well as serotype based on data available on the MLST database (http://mlst.warwick.ac.uk/mlst/dbs/Senterica/)

Differences in proportions were compared using the chi‐square test and Fisher's exact test, when the chi‐square test was not relevant. A *P*‐value <0.05 was considered statistically significant.

## Results

Of 204 chicken farms, 93 (45.6%, 95% CI = 38.8–52.4%) tested positive for NTS. There was no statistically significant difference in the NTS farm‐level prevalence between household farms (46/102, 45.1%) and small farms (47/102, 46.1%). NTS was recovered from nine chicken farmers (4.4%, 95% CI = 1.6–7.2%), six rural subjects (2.9%, 95% CI = 0.6–5.3%) and two urban subjects (2.0%, 95% CI = 0.0–4.7%). The prevalence of NTS did not statistically differ between chicken farmers and unexposed individuals. The prevalence of NTS among farmers of NTS‐positive chicken flocks (5/93, 5.4%; 95% CI = 0.8–10.0%) was similar to the prevalence of NTS among farmers of NTS‐negative chicken flocks (4/111, 3.6%; 95% CI = 0.1–7.1%).

**Figure 1:**
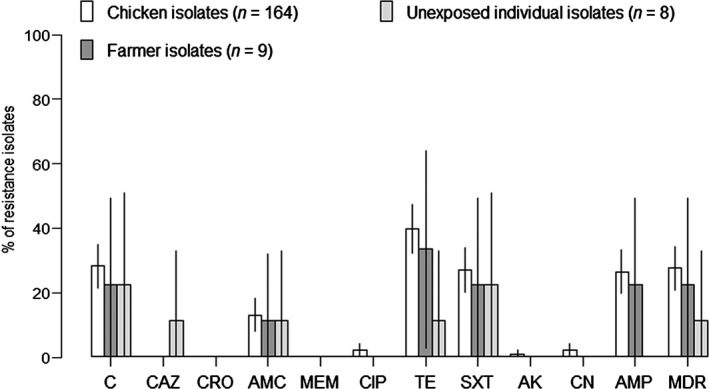
Percentage of NTS isolates resistant to a panel of 11 antimicrobials. C: chloramphenicol (30 *μ*g), CAZ: ceftazidime (30 *μ*g), CRO: ceftriaxone (30 *μ*g), AMC: amoxicilin/clavulanic acid (30 *μ*g), MEM: meropenem (10 *μ*g), CIP: ciprofloxacin (5 *μ*g), TE: tetracycline (30 *μ*g), SXT: trimethoprim‐sulphamethoxazole (10 *μ*g), AK: amikacin (30 *μ*g), CN: gentamicin (10 *μ*g), AMP: ampicillin (10 *μ*g), MDR: Multidrug resistance (resistant against at least three classes of antimicrobial).

Among 164 chicken NTS isolates, the highest observed levels of resistance were against tetracycline (39.6%), chloramphenicol (28.0%), sulfamethoxazole‐trimethoprim (26.8%), ampicillin (26.2%) and amoxicillin plus clavulanic acid (12.8%). The proportion of strains resistant against ciprofloxacin and gentamicin was 1.8%. No resistance against meropenem, ceftazidime and ceftriaxone was observed. A total of nine NTS isolates from farmers and eight NTS isolates from individuals unexposed to chicken farming were tested for antimicrobial resistance, which indicated similar levels of resistance against all tested antimicrobials. 27.4% (45/164), 22.2% (2/9) and 12.5% (1/8) of NTS isolates from chickens, farmers and unexposed individuals were multidrug resistant, respectively (Fig. 1). Interestingly, one of the two multidrug‐resistant isolates from farmers was identical to the most common resistance pattern found in chicken flocks (chloramphenicol – ampicillin – tetracycline – trimethoprim/sulfamethoxazole) (Data not shown).

Multilocus sequence typing was performed on 163 of 164 chicken isolates from 93 chicken farms (one isolate could not be recovered after storage) and on all 17 isolates from humans. *Salmonella* Weltevreden was the most common serovar detected in chicken farms (10.3% of farms), farmers (2.0%), rural subjects (1.0%) and urban subjects (2.0%). Besides *Salmonella* Weltevreden, the predominant serovars in chickens were *Salmonella* Enteritidis, *Salmonella* Paratyphi B var Java monophasic, *Salmonella* Albany, *Salmonella* Derby, *Salmonella* Give, *Salmonella* Newport and *Salmonella* Typhimurium. *Salmonella* Enteritidis and *Salmonella* Typhimurium were only found in chickens and were detected in 4.4% and 1.5% of the chicken farms, respectively (Table 1).

**Table 1:** Distribution of different serovars of NTS isolated from chickens and humans in southern Vietnam

NTS serovar	No. culture positive (%)			
	Chicken farms^a^ (*n* = 204)	Chicken farmers (*n* = 204)	Rural subjects (*n* = 204)	Urban subjects (*n* = 102)
Weltevreden	21 (10.3)	4 (2.0)	2 (1.0)	2 (2.0)
Enteritidis	9 (4.4)	0	0	0
Paratyphi B var Java monophasic	9 (4.4)	0	0	0
Albany	7 (3.4)	0	0	0
Derby	6 (2.9)	0	1 (0.5)	0
Give	6 (2.9)	2 (1.0)	0	0
Newport	4 (2.0)	0	1 (0.5)	0
Typhimurium	3 (1.5)	0	0	0
Braenderup	1 (0.5)	1 (0.5)	0	0
Orientalis	0	1 (0.5)	0	0
Rubislaw	0	1 (0.5)	0	0
Ohio	0	0	1 (0.5)	0
Other serovars^b^	28 (13.7)	0	0	0
Untypeable	19 (9.3)	0	0	0
Any serovar	93 (45.6)	9 (4.4)	6 (2.9)	2 (2.0)

a On 19 farms, multiple serovars were present.

b Other serovars: Anatum, Senftenberg, Stanley, Virchow (each serovar was present on 3 farms); Kentucky, London, Montevideo, Typhimurium monophasic (each serovar was present on 2 farms); Cerro, Indian, Litchfield, Mbandaka, Meleagridis, Oslo, Poona, Tennessee (each serovar was present on one farm).

Non‐typhoidal *Salmonella enterica* were detected in both chickens and farmers on five of 204 farms (2.5%, 95% CI = 0.3–4.6%). MLST revealed that the serovar of NTS isolates obtained from the farmer and their chicken on the same farm were identical in one farm (*Salmonella* Weltevreden), but differed between farmer and their chickens for the other four farms (Table 2).

**Table 2:** Serovar and antimicrobial resistance pattern of NTS isolated from chicken flocks and farmers from the same farm

Farm ID	Source	Isolate number	*Salmonella* serovar	Antimicrobial resistance pattern^a^
CG 37	Farmer	1	Weltevreden	Fully susceptible
	Chicken	1	Untypeable	Fully susceptible
CT 67	Farmer	1	Rubislaw	C‐AMC‐TE‐AMP
	Chicken	1	Albany	Fully susceptible
MT 26	Farmer	1	Weltevreden	Fully susceptible
	Chicken	1	Weltevreden	Fully susceptible
	Chicken	2	Weltevreden	Fully susceptible
MT 28	Farmer	1	Give	C‐TE‐SXT‐AMP
	Chicken	2	Enteritidis	TE
MT 53	Farmer	1	Weltevreden	Fully susceptible
	Chicken	1	Senftenberg	CIP‐TE‐SXT
	Chicken	2	Senftenberg	Fully susceptible
	Chicken	3	Cerro	Fully susceptible

a Isolates were tested for susceptibility to 11 antimicrobials using dick diffusion method and interpreted according to breakpoints as defined by Clinical and Laboratory Standard Institute (11). C: chloramphenicol (30 *μ*g), AMC: amoxicillin/clavulanic acid (30 *μ*g), CIP: ciprofloxacin (5 *μ*g), TE: tetracycline (30 *μ*g), SXT: trimethoprim‐sulphamethoxazole (10 *μ*g), AMP: ampicillin (10 *μ*g).

## Discussion

To our knowledge, this is the first field survey reporting on prevalence of asymptomatic NTS colonization in humans occupationally exposed and unexposed to chickens in Vietnam. The observed prevalence of asymptomatic NTS colonization in humans was 3.3%, a figure considerably higher than the reported prevalence of asymptomatic NTS in developed countries (0.3–0.4%) (Hellard et al., 2000; de Wit et al., 2001; Nataro et al., 2006). However, our results were similar to the results from a study performed in 2004 in Hanoi, Vietnam (3.1%) (Do et al., 2007) and from Thailand in 2003 (4.7%) (Sirinavin et al., 2004).

We found a higher prevalence of NTS colonization among chicken farmers (4.4%) compared with unexposed individuals (2.6%). The prevalence of NTS colonization in the rural subjects was also higher (2.9%) than in the urban subjects (2.0%). Similarly, farmers of NTS‐positive chicken flocks had a higher prevalence of infection compared with farmers of NTS‐negative chicken flocks (5.4% versus 3.6%). However, none of these differences were statistically significant.

Our study demonstrated a high prevalence (45.6%) and high diversity of NTS serovars in both household‐size and small‐size chicken farms in the Mekong Delta of Vietnam, similar to the results from a recent survey (64.7%) carried out in Dong Thap, another Mekong Delta province (Tu et al., 2015). In unconfined flocks, swabs may potentially gathered faecal material from other animals in the farm. However, in those cases, sampling was carried out near the perching and eating areas where chicken droppings were visible, so it is expected that the overwhelming majority of faecal material and *Salmonella* strains were of chicken origin. In terms of antimicrobial sensitivity, multidrug resistance was commonly observed in the chicken NTS isolates. Of antimicrobials of critical importance, the prevalence of resistance against ampicillin was particularly high among tested isolates (26.2%). However, levels of resistance against aminoglycosides (gentamicin) and fluoroquinolones (ciprofloxacin) were low (<2%).

We acknowledge several limitations in our study. Firstly, the number of NTS isolates from humans was small, limiting the power to demonstrate any statistical difference between study cohorts. Secondly, the cross‐sectional study design may preclude the demonstration of transmission of any particular serovar, which is highly depending on the dynamics of NTS transmission between chickens and humans. It is therefore possible that the presence of NTS in the farmer results from earlier infection and further clearance in the current chicken flock, or from transmission from a previous flock. In addition, differences in NTS isolation methods for chicken and human samples may have had an impact on the sensitivity of detection. A higher sensitivity of detection of the ISO 6572: 2002 (Annex D) method compared with the WHO method for human samples is to be expected, as the former include a pre‐enrichment, selective enrichment phase, which allows the detection of low numbers of *Salmonella* such as those likely to be found in asymptomatic chicken samples (Carrique‐Mas and Davies, 2008).

In spite of these limitations, we found that one of 21 (4.7%) of the farmers with *Salmonella* Weltevreden infected chicken flocks was also *Salmonella* Weltevreden positive compared with three of 183 (1.6%) of the farmers without *Salmonella* Weltevreden infected chicken flocks. Interestingly, the *Salmonella* Weltevreden isolated from the farmer had an identical antimicrobial resistance pattern to the isolate from his or her chickens (fully susceptible). It has been suggested that *Salmonella* Weltevreden may have acquired properties which facilitate adaptation to a broader range of hosts (Brankatschk et al., 2012), and *Salmonella* Weltevreden has been shown to be able to persist in manure and soil for prolonged periods of time (Arthurson et al., 2011).

We believe that our study reflects the epidemiological situation of NTS in the Mekong delta of Vietnam, characterized by a high prevalence of infection in chicken flocks and a relatively high prevalence of colonization of human adults. Our study also underscores the need for additional larger and preferably longitudinal studies to investigate transmission dynamics of NTS between and within animal and human host populations.
